# Supplementary figures and images for: Can we measure beauty? Computational evaluation of coral reef aesthetics
Source: PeerJ. 2015 Nov 10;3:e1390. doi: 10.7717/peerj.1390 (PMC4647610; doi:10.7717/peerj.1390)

Relative Importance

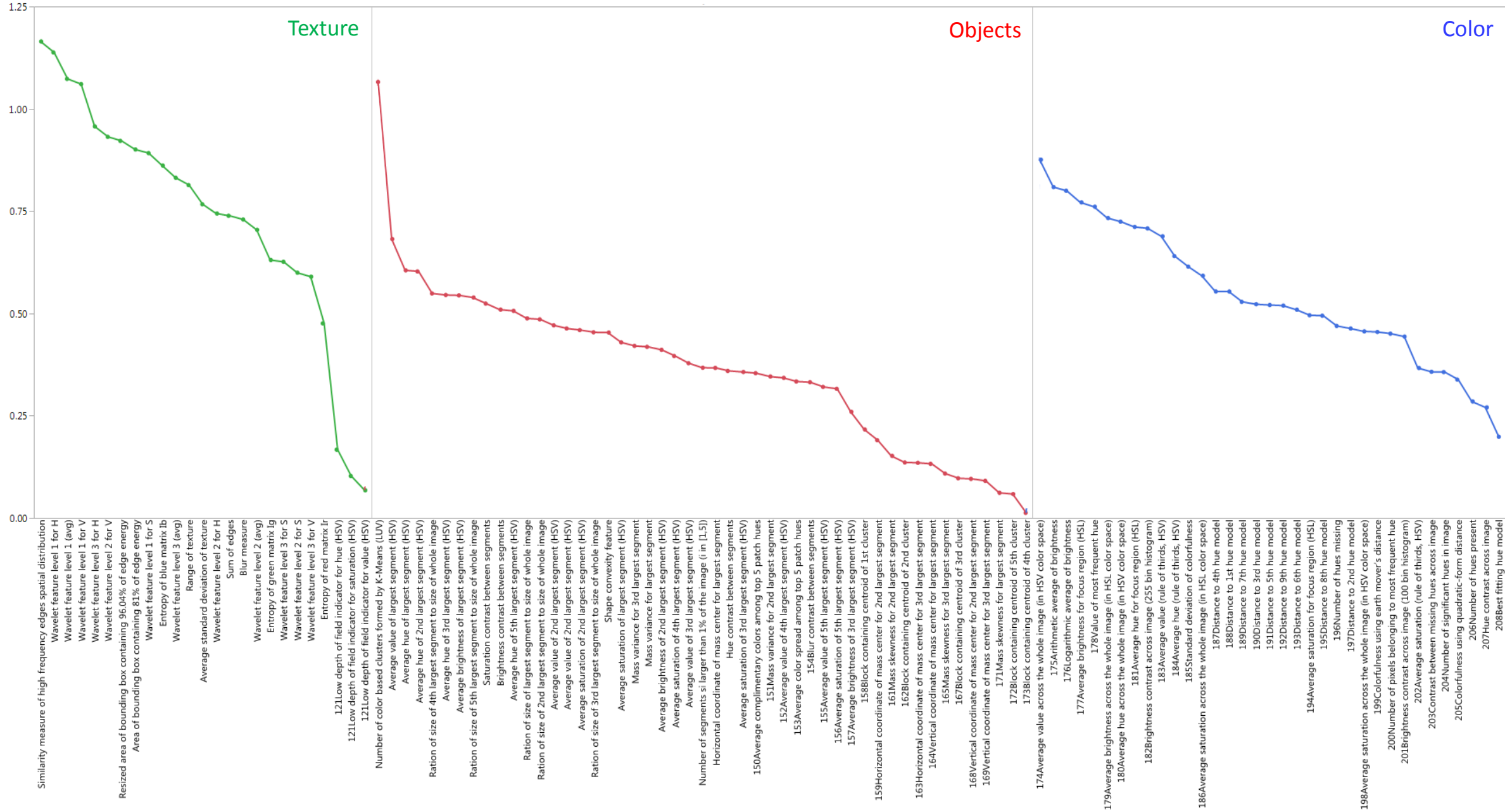

Supplement: Figure S1 — Relative importance of all 109 features derived from a random forest approach. Features are grouped into three general feature groups, texture of the entire image (texture), color and brightness of the entire image (color), and size, color and brightness, and distribution of objects within the image (objects). [file peerj-03-1390-s001.pdf]

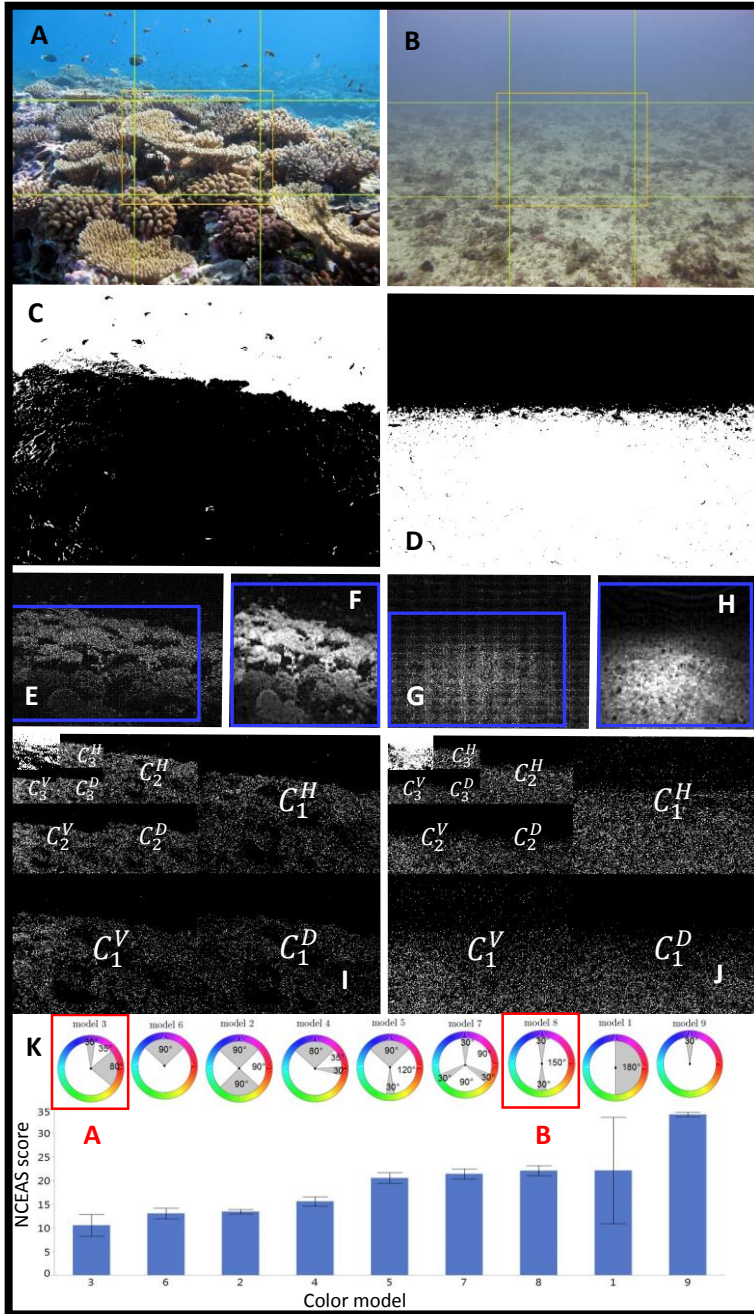

Supplement: Figure S2 — Example pictures of a healthy (left) and a degraded reef (right). The applied measurements for brightness contrast across the whole image f28 shows 97 for (A) and 47 for (B). The green lines depict the central focus region which outlines the segment of interest used for ‘Rule of Third’ features. The orange line marks the Focus region used for features f55 through f56, where an additional margin (μ = 0.1) has been included. (C) and (D) show pictures after segmentation by K means, using K = 2 and m = 1. From these images the number of connected components can be calculated by implementing feature f58 (C = 1,470, D = 2,369). (E) and (G) show the Laplacian image produced for feature f30, (F) and (H) show the resized and normalized Laplacian image which serves as basis for the calculation of f31. The blue bounding boxes contain 81% (E = 0.623, G = 0.611) and 96.04% (F = 0.089, H = 0.079) of the edge energy respectively. (I) and (J) show the images after a three-level wavelet transform performed on the saturation channel IS. K gives an overview of color models used to compare the analyzed images, or objects within images against. The bar chart shows the average NCEAS score where pictures matching to the respective color model were taken. The red boxes indicate the model that fits best to image A and B respectively. [file peerj-03-1390-s002.pdf]
