# Supplementary material for: Can we measure beauty? Computational evaluation of coral reef aesthetics
Source: PeerJ. 2015 Nov 10;3:e1390. doi: 10.7717/peerj.1390 (PMC4647610; doi:10.7717/peerj.1390)
Supplement: Supplemental Information 1 — Matlab script to extract 109 aesthetic features. [file peerj-03-1390-s003.docx]

function featuresExtraction(myfolder)

% featuresExtraction(folder)

% Calculates all the features for all the images in the folder 'myfolder',

% and saves them in a .mat file called 'features_myfolder',

% which contains a matrix with the features for all our images 'collected_features', and a matrix 'names' containing the list of names of the pictures analysed.

%

% /!\ We will need to load the Laplacian images Mp and Ms used with the function edgeDistance

% You first have to calculate those images using generate_mean and save

% those two matrices in a file called laplacianMean.mat

%list=ls('myfolder') %just used to print a clear list of the image in the file

list=dir(myfolder)

nb_data=size(list,1)-2;

names=cell(1,nb_data);

% initialization of the features

fprintf('Initialization of the features \n');

h=zeros(nb_data,1);

s=zeros(nb_data,1);

v=zeros(nb_data,1);

s_=zeros(nb_data,1);

l_=zeros(nb_data,1);

hist_distance=zeros(nb_data,1);

emd_distance=zeros(nb_data,1);

freq_hue=zeros(nb_data,1);

dev_color=zeros(nb_data,1);

nbHue=zeros(nb_data,1);

hueContrast=zeros(nb_data,1);

missingHue=zeros(nb_data,1);

missingContrast=zeros(nb_data,1);

maxPixel=zeros(nb_data,1);

hue_count=zeros(nb_data,1);

modelDistance=[];

hue_model=zeros(nb_data,1);

arithmBrightness=zeros(nb_data,1);

logarithBrightness=zeros(nb_data,1);

brightnessContrast=zeros(nb_data,1);

contrast_quality=zeros(nb_data,1);

bounding_area_ratio=zeros(nb_data,1);

edge_quality=zeros(nb_data,1);

bounding_quality=zeros(nb_data,1);

sum_edges=zeros(nb_data,1);

texture_range=zeros(nb_data,1);

texture_deviation=zeros(nb_data,1);

entropy_r=zeros(nb_data,1);

entropy_g=zeros(nb_data,1);

entropy_b=zeros(nb_data,1);

TextureH1=zeros(nb_data,1);

TextureH2=zeros(nb_data,1);

TextureH3=zeros(nb_data,1);

TextureS1=zeros(nb_data,1);

TextureS2=zeros(nb_data,1);

TextureS3=zeros(nb_data,1);

TextureV1=zeros(nb_data,1);

TextureV2=zeros(nb_data,1);

TextureV3=zeros(nb_data,1);

TextureAvgH=zeros(nb_data,1);

TextureAvgS=zeros(nb_data,1);

TextureAvgV=zeros(nb_data,1);

Low_DOFH=zeros(nb_data,1);

Low_DOFS=zeros(nb_data,1);

Low_DOFV=zeros(nb_data,1);

global_blur=zeros(nb_data,1);

h3=zeros(nb_data,1);

s3=zeros(nb_data,1);

v3=zeros(nb_data,1);

focus_hue=zeros(nb_data,1);

focus_saturation=zeros(nb_data,1);

focus_lightness=zeros(nb_data,1);

XY_100_=zeros(nb_data,1);

numb_conncomp=zeros(nb_data,1);

AvgH=[];

AvgS=[];

AvgV=[];

SI_XY_=[];

Centroid=[];

Color_spread=zeros(nb_data,1);

Complem_colors=zeros(nb_data,1);

Convexity=zeros(nb_data,1);

Centroid_x=[];

Centroid_y=[];

Shape_variance=[];

Shape_skewness=[];

Segment_brightness=[];

Hue_contrast=zeros(nb_data,1);

Saturation_contrast=zeros(nb_data,1);

Brightness_contrast=zeros(nb_data,1);

Blur_contrast=zeros(nb_data,1);

%Loading the Laplacian images Mp and Ms used with the function edgeDistance

%You first have to calculate those images using laplacianMean and save

%those two matrices in a file called laplacianMean.mat

fprintf('Loading laplacianMean');

load laplacianMean

% /!\ the for loop starts at 3 (4 for mac)because list(1).name='.' and list(2).name='..'

fprintf('Feature extraction \n');

for image=3:size(list)

tic

i=image-2;

string=strcat(myfolder,'/',list(image).name)

names{i}=list(image).name;

Irgb=imread(string);

Irgb=im2double(Irgb);

Ir=Irgb(:,:,1);

Ig=Irgb(:,:,2);

Ib=Irgb(:,:,3);

Ihsv=rgb2hsv(Irgb);

Ih=Ihsv(:,:,1);

Is=Ihsv(:,:,2);

Iv=Ihsv(:,:,3);

Ihsl=rgb2hsl(Irgb);

Ih_=Ihsl(:,:,1);

Is_=Ihsl(:,:,2);

Il_=Ihsl(:,:,3);

%average hue, saturation, value (HSV)

h(i)=mean2(Ih);

s(i)=mean2(Is);

v(i)=mean2(Iv);

%average saturation and lightness (HSL)

s_(i)=mean2(Is_);

l_(i)=mean2(Il_);

%colorfulness

[hist_distance(i),emd_distance(i)]=rgbCubes(Ir,Ig,Ib);

%most frequent hue and standard deviation of colorfulness

Ihb=findReplace(Ih,Ihsl);

idx=Ihb>0;

freq_hue(i)=mode(mode(Ihb(idx)));

dev_color(i)=std(var(Ihb));

%Number of distinct hues present and missing in the image, their

%contrast and number of pixels that belong to the most frequent hue

n=20;

C=0.1;

c=0.01;

[~, nbHue(i), hueContrast(i), missingHue(i), missingContrast(i), maxPixel(i)]=hueHistogram(Ihsl,n,C,c);

%Hue count (re-using hueHistogram with a different color space and C)

n=20;

C=0.05;

c=0.01;

[~,Hue_count,~,~,~,~]=hueHistogram(Ihsv,n,C,c);

hue_count(i)=n-Hue_count;

fprintf('Hue model fitting \n');

%hue models fitting

model_threshold=10;

modelNormalDistance=zeros(9,1);

for k=1:9

g=@(alpha)hueModel(Ih_,Is_,alpha,k);

alpha0=fminbnd(g,0,360);

[normalizedDistance]=hueModel(Ih_,Is_,alpha0,k);

modelNormalDistance(k,1)=normalizedDistance;

end

modelDistance=[modelDistance;transpose(modelNormalDistance)];

model=modelNormalDistance.*(modelNormalDistance<model_threshold);

if sum(model)>0

hue_model(i)=max(find(model));

else

[~,hue_model(i)]=min(modelNormalDistance);

end

% arithmetic and logarithmic average brightness + brightness contrast

[arithmBrightness(i),logarithBrightness(i),brightnessContrast(i)]=brightness(Ir,Ig,Ib);

%Contrast quality

[contrast_quality(i)]=brightnessHistogram(Ir,Ig,Ib);

fprintf('Getting bounding_area_ratio \n');

%Edge distribution metric

[bounding_area_ratio(i)]=bounding_box(Ir,Ib,Ig);

%spatial distribution of the high frequency edges and area of the bounding box

[edge_quality(i),bounding_quality(i)]=edgeDistance(Ir,Ig,Ib,Mp,Ms);

%sum of edges

sum_edges(i)=edgeSum(Ir,Ig,Ib);

%range of texture

a=rangefilt(Ihsv);

texture_range(i)=sum(mean2(a))/3;

%standard deviation of texture

b=stdfilt(Ihsv);

texture_deviation(i)=sum(mean2(b))/3;

%entropy of the red, green and blue matrices

entropy_r(i)=entropy(Ir);

entropy_g(i)=entropy(Ig);

entropy_b(i)=entropy(Ib);

fprintf('Getting the wavelet related features \n');

%wavelet related features

[texture,low_DOF]=waveletTexture(Ihsl);

TextureH1(i)=texture(1,1);

TextureH2(i)=texture(2,1);

TextureH3(i)=texture(3,1);

TextureS1(i)=texture(1,2);

TextureS2(i)=texture(2,2);

TextureS3(i)=texture(3,2);

TextureV1(i)=texture(1,3);

TextureV2(i)=texture(2,3);

TextureV3(i)=texture(3,3);

TextureAvgH(i)=texture(4,1);

TextureAvgS(i)=texture(4,2);

TextureAvgV(i)=texture(4,3);

Low_DOFH(i)=low_DOF(1);

Low_DOFS(i)=low_DOF(2);

Low_DOFV(i)=low_DOF(3);

fprintf('Getting the blur feature \n');

%blur measure

[global_blur(i)]=gaussian_blur(Ir,Ig,Ib);

%averages from rule of thirds

margin=0;

h3(i)=thirdsAvg(Ih,margin);

s3(i)=thirdsAvg(Is,margin);

v3(i)=thirdsAvg(Iv,margin);

%Average hue, saturation and lightness of the focus region

margin=0.1;

focus_hue(i)=thirdsAvg(Ih_,margin);

focus_saturation(i)=thirdsAvg(Is_,margin);

focus_lightness(i)=thirdsAvg((Ir+Ig+Ib)/3,margin);

fprintf('Getting all the segmentation related features \n');

%all the region composition features (ie the segmentation related

%features)

k=2;

m=1;

[nb_cc,avgH,avgS,avgV,XY_100,SI_XY,centroid,color_spread,complem_colors,convexity,centroid_x,centroid_y,shape_variance,shape_skewness,lightness,hue_contrast,saturation_contrast,brightness_contrast,blur_contrast]=seg1(Irgb,k,m);

XY_100_(i)=XY_100;

numb_conncomp(i)=nb_cc;

AvgH=[AvgH; transpose(avgH)];

AvgS=[AvgS; transpose(avgS)];

AvgV=[AvgV; transpose(avgV)];

SI_XY_=[SI_XY_; transpose(SI_XY)];

Color_spread(i)=color_spread;

Complem_colors(i)=complem_colors;

Centroid=[Centroid; transpose(centroid)];

Convexity(i)=convexity;

Centroid_x=[Centroid_x; transpose(centroid_x)];

Centroid_y=[Centroid_y; transpose(centroid_y)];

Shape_variance=[Shape_variance; transpose(shape_variance)];

Shape_skewness=[Shape_skewness; transpose(shape_skewness)];

Segment_brightness=[Segment_brightness; transpose(lightness)];

Hue_contrast(i)=hue_contrast;

Saturation_contrast(i)=saturation_contrast;

Brightness_contrast(i)=brightness_contrast;

Blur_contrast(i)=blur_contrast;

toc

end

collected_features=[h s v s_ l_ hist_distance emd_distance freq_hue dev_color nbHue missingHue hueContrast missingContrast maxPixel hue_count modelDistance hue_model arithmBrightness logarithBrightness brightnessContrast contrast_quality bounding_area_ratio bounding_quality edge_quality sum_edges texture_range texture_deviation entropy_r entropy_g entropy_b TextureH1 TextureH2 TextureH3 TextureS1 TextureS2 TextureS3 TextureV1 TextureV2 TextureV3 TextureAvgH TextureAvgS TextureAvgV global_blur h3 s3 v3 focus_hue focus_saturation focus_lightness numb_conncomp XY_100_ SI_XY_ Centroid AvgH AvgS AvgV Segment_brightness Color_spread Complem_colors Centroid_x Centroid_y Shape_variance Shape_skewness Convexity Hue_contrast Saturation_contrast Brightness_contrast Blur_contrast Low_DOFH Low_DOFS Low_DOFV];

save(strcat('features_','Carysfort'),'collected_features','names');

%%%%%%%%%%%%%%%%%%%%%%%%%%%%%%%%%%%%%%%%%%%%%%%%%%%%%%%

function [bounding_area_ratio]=bounding_box(Ir,Ib,Ig)

% [bounding_area_ratio]=bounding_box(Ir,Ib,Ig)

% Considering a picture whose red, green and blue channels are given in Ir,

% Ig and Ib, edgeDistance computes its laplacian image, and returns the

% ratio of the area of the bounding box containing 81% of the edge energy,

% and the area of the photo

h = fspecial('laplacian', 0.2);

IR=abs(imfilter(Ir,h,'replicate'));

IG=abs(imfilter(Ig,h,'replicate'));

IB=abs(imfilter(Ib,h,'replicate'));

edge_img=(IR+IG+IB)/3;

percentage=0.9;

[a1,b1]=find_energy(percentage,sum(edge_img,2),size(edge_img,1));

[a2,b2]=find_energy(percentage,sum(edge_img,1),size(edge_img,2));

bounding_area_ratio=(b1-a1+1)*(b2-a2+1)/(size(edge_img,1)*size(edge_img,2));

%%%%%%%%%%%%%%%%%%%%%%%%%%%%%%%%%%%%%%%%%%%%%%%%%%%%%%%

function [ arithmBrightness,logarithmBrightness, brightnessContrast ] = brightness( Ir,Ig,Ib )

%[ arithmBrightness,logarithmBrightness, brightnessContrast ] = brightness( Ir,Ig,Ib )

% Calculates the average arithmetic brightness, the average logarithmic

% brightness, and a brightness contrast using a 100 bins histogram

% note: need Ir,Ig,Ib to have values in [0;1]

%arithmetic average brightness

M=(Ir+Ig+Ib)/3;

arithmBrightness=mean2(M);

%logarithmic average brightness + brightness histogram

sum=0;

epsilon=0.001;

B=zeros(100,1);

N=100*M;

for m=1:size(Ir,1)

for n=1:size(Ir,2)

sum = sum + log(epsilon + M(m,n));

if (N(m,n)~=100)

B(floor(N(m,n))+1)=B(floor(N(m,n))+1)+1;

else

B(100)=B(100)+1;

end

end

end

logarithmBrightness=exp(sum/(size(Ir,1)*size(Ir,2)));

[C,I]=max(B);

area=C;

a=I;

b=I;

nbPixel=size(Ir,1)*size(Ir,2);

while (area/nbPixel)<0.98

if a>1

a=a-1;

area=area+B(a);

end

if b<100

b=b+1;

area=area+B(b);

end

end

brightnessContrast=b-a+1;

end

%%%%%%%%%%%%%%%%%%%%%%%%%%%%%%%%%%%%%%%%%%%%%%%%%%%%%%%

function [contrast_quality]=brightnessHistogram(Ir,Ig,Ib)

%[contrast_quality]=brightnessHistogram(Ir,Ig,Ib)

% Returns the width of the smallest region containing 0.98percent of the

% brightness histogram of the image

Ir=255*Ir;

Ig=255*Ig;

Ib=255*Ib;

Hr=zeros(256,1);

Hg=zeros(256,1);

Hb=zeros(256,1);

for i=1:size(Ir,1)

for j=1:size(Ir,2)

Hr(1+floor(Ir(i,j)))=Hr(1+floor(Ir(i,j)))+1;

Hg(1+floor(Ig(i,j)))=Hg(1+floor(Ig(i,j)))+1;

Hb(1+floor(Ib(i,j)))=Hb(1+floor(Ib(i,j)))+1;

end

end

H=(Hr+Hg+Hb)/(size(Ir,1)*size(Ir,2));

percentage=0.98;

[a,b]=find_energy(percentage,H,256);

contrast_quality=b-a;

end

%%%%%%%%%%%%%%%%%%%%%%%%%%%%%%%%%%%%%%%%%%%%%%%%%%%%%%%

function [IH]=context(Ih,m)

% [IH]=context(Ih,m)

% Performs a uniform blur on Ih, but independantly of its size, with

% ones(m) as a kernel matrix

% Ih must be a 2D image, m an integer >0

if m>1

if size(Ih,1)>size(Ih,2)

m1=floor(m*size(Ih,1)/3072);

m2=floor(m*size(Ih,2)/2304);

else

m1=floor(m*size(Ih,1)/2304);

m2=floor(m*size(Ih,2)/3072);

end

%sums the i,...,i+m1-1 lines of Ih

sum_Ih=[zeros(m1-1,size(Ih,2));Ih];

for i=1:m1-1

sum_Ih=sum_Ih+[zeros(m1-i-1,size(Ih,2));Ih;zeros(i,size(Ih,2))];

end

%sums the i,...,i+m2-1 columns of sum_Ih

sum_Ih2=[zeros(size(Ih,1)+m1-1,m2-1),sum_Ih];

for i=1:m2-1

sum_Ih2=sum_Ih2+[zeros(size(Ih,1)+m1-1,m2-i-1) sum_Ih zeros(size(Ih,1)+m1-1,i)];

end

%suppress the additional lines and columns

for i=1:m1-1

sum_Ih2(1,:)=[];

sum_Ih2(size(sum_Ih2,1),:)=[];

end

for i=1:m2-1

sum_Ih2(:,1)=[];

sum_Ih2(:,size(sum_Ih2,2))=[];

end

%we have added the value of m1*m2 pixels, so to have an average, we have to

%divide it by m1*m2

IH=sum_Ih2/(m1*m2);

else

IH=Ih;

end

end

%%%%%%%%%%%%%%%%%%%%%%%%%%%%%%%%%%%%%%%%%%%%%%%%%%%%%%%

function [edge_quality,bounding_quality]=edgeDistance(Ir,Ig,Ib,Mp,Ms)

% [edge_quality,bounding_quality]=edgeDistance(Ir,Ig,Ib,Mp,Ms)

%

% Considering a picture whose red, green and blue channels are given in Ir,

% Ig and Ib, edgeDistance computes its normalized 100x100 laplacian image,

% and compares it to Mp and Ms.

% Mp and Ms have to be to be two normalized 100x100 laplacian images also

% (they are supposed to be the mean Laplacian image of the good (resp. bad)

% photos). They can be constructed using the function laplacianMean.

% edge_quality=ds-dp , where ds (resp dp) is the distance between this

% laplacian image and Ms (resp Mp)

%

% bounding_quality is 1-the area of the bounding box containing 96.04% of

% the edge energy

%Mp and Ms are the mean Laplacian image of the good and bad photos, respectively

[laplacian_img]=laplacianImage(Ir,Ig,Ib);

DP=laplacian_img-Mp;

DS=laplacian_img-Ms;

dp=norm(DP(:),1);

ds=norm(DS(:),1);

edge_quality=ds-dp;

percentage=0.98;

[a1,b1]=find_energy(percentage,sum(laplacian_img,2),size(laplacian_img,1));

[a2,b2]=find_energy(percentage,sum(laplacian_img,1),size(laplacian_img,2));

bounding_quality=1-(b1-a1+1)*(b2-a2+1)/10000;

%%%%%%%%%%%%%%%%%%%%%%%%%%%%%%%%%%%%%%%%%%%%%%%%%%%%%%%

function [ a ] = edgeSum( Ir,Ig,Ib)

%[ a ] = edgeSum( Ir,Ig,Ib)

% x=sum(sum(edge(Ir)))/(size(Ir,1)*size(Ir,2));

% y=sum(sum(edge(Ig)))/(size(Ig,1)*size(Ig,2));

% z=sum(sum(edge(Ib)))/(size(Ib,1)*size(Ib,2));

%

% a=((x+y+z)/3);

x=sum(sum(edge(Ir)))/(size(Ir,1)*size(Ir,2));

y=sum(sum(edge(Ig)))/(size(Ig,1)*size(Ig,2));

z=sum(sum(edge(Ib)))/(size(Ib,1)*size(Ib,2));

a=((x+y+z)/3);

end

%%%%%%%%%%%%%%%%%%%%%%%%%%%%%%%%%%%%%%%%%%%%%%%%%%%%%%%

function [distance2]=emd(H,D,n)

nbBox=n^3;

nbBox2=nbBox*nbBox;

A=zeros(2*nbBox,nbBox2);

f=zeros(1,nbBox2);

for i=1:nbBox

for j=1:nbBox

A(i,j+nbBox*(i-1))=1;

A(i+nbBox,i+nbBox*(j-1))=1;

f(1,j+nbBox*(i-1))=D(i,j);

end

end

b=ones(2*nbBox,1)/nbBox;

for i=1:nbBox

b(i)=H(i);

end

Aeq=ones(1,nbBox2);

beq=min( norm(H,1),1);

lb=zeros(1,nbBox2);

ub=[];

[~,distance2] = linprog(f,A,b,Aeq,beq,lb,ub);

end

%%%%%%%%%%%%%%%%%%%%%%%%%%%%%%%%%%%%%%%%%%%%%%%%%%%%%%%

function [a,b]=find_energy(percentage,vector,n)

%finds the indice a and b, where b-a is the smallest distance with still

%sum_{k=a}^{b}vector(k)>0.9*sum_{k=1}^{size(vector)}vector(k)

%/!\ n is the size of vector

if percentage>1 || percentage<0

error('percentage must be in [0,1]');

end

total=norm(vector,1);

inverse_percent=(1-percentage)*total;

left_limit=0;

content=0;

while content<inverse_percent

left_limit=left_limit+1;

content=content+vector(left_limit);

end

right_limit=0;

content=0;

while content<inverse_percent

content=content+vector(n-right_limit);

right_limit=right_limit+1;

end

a=1;

b=n;

percent=percentage*total;

for i=1:left_limit

for j=0:right_limit-1

s=0;

for k=i:n-j

s=s+vector(k);

end

if (s>=percent)&&(n-j-i<b-a)

a=i;

b=n-j;

end

end

end

%%%%%%%%%%%%%%%%%%%%%%%%%%%%%%%%%%%%%%%%%%%%%%%%%%%%%%%

function [ Ih ] = findReplace( Ih, Ihsl )

%[ Ih ] = findReplace( Ih, Ihsl )

%Returns the matrix Ih, where all the pixels with a saturation lower than

%0.2 or a lightness out of ]0.15,0.95[ have been set to 0.

% if Ihsl(i,j,2)<=0.2 || (Ihsl(i,j,3)>=0.95 || Ihsl(i,j,3)<=0.15)

% Ih(i,j)=0;

% end

for i=1:size(Ih,1)

for j=1:size(Ih,2)

if Ihsl(i,j,2)<=0.2 || (Ihsl(i,j,3)>=0.95 || Ihsl(i,j,3)<=0.15)

Ih(i,j)=0;

end

end

end

end

%%%%%%%%%%%%%%%%%%%%%%%%%%%%%%%%%%%%%%%%%%%%%%%%%%%%%%%

function [blur]=gaussian_blur(Ir,Ig,Ib)

% [blur]=gaussian_blur(Ir,Ig,Ib)

% using theta=0.45

% blur is in [-1; 0], -1 being completely blurred, and 0 a very sharp image

I_blurred=(Ir+Ig+Ib)/3;

M=size(I_blurred,1);

N=size(I_blurred,2);

Y=fft2(I_blurred)/sqrt(M*N);

theta=0.45;

abs_Y=abs(Y);

[row,column]=find(abs_Y>theta);

select_row=row<M/2;

select_column=column<N/2;

m=max(row.*select_row);

n=max(column.*select_column);

blur=max(2*(m-floor(M/2))/M,2*(n-floor(N/2))/N);

end

%%%%%%%%%%%%%%%%%%%%%%%%%%%%%%%%%%%%%%%%%%%%%%%%%%%%%%%

function rgb=hsl2rgb(hsl_in)

%Converts Hue-Saturation-Luminance Color value to Red-Green-Blue Color value

%

%Usage

% RGB = hsl2rgb(HSL)

%

% converts HSL, a M [x N] x 3 color matrix with values between 0 and 1

% into RGB, a M [x N] X 3 color matrix with values between 0 and 1

%

%See also rgb2hsl, rgb2hsv, hsv2rgb

% (C) Vladimir Bychkovsky, June 2008

% written using:

% - an implementation by Suresh E Joel, April 26,2003

% - Wikipedia: http://en.wikipedia.org/wiki/HSL_and_HSV

hsl=reshape(hsl_in, [], 3);

H=hsl(:,1);

S=hsl(:,2);

L=hsl(:,3);

lowLidx=L < (1/2);

q=(L .* (1+S) ).*lowLidx + (L+S-(L.*S)).*(~lowLidx);

p=2*L - q;

hk=H; % this is already divided by 360

t=zeros([length(H), 3]); % 1=R, 2=B, 3=G

t(:,1)=hk+1/3;

t(:,2)=hk;

t(:,3)=hk-1/3;

underidx=t < 0;

overidx=t > 1;

t=t+underidx - overidx;

range1=t < (1/6);

range2=(t >= (1/6) & t < (1/2));

range3=(t >= (1/2) & t < (2/3));

range4= t >= (2/3);

% replicate matricies (one per color) to make the final expression simpler

P=repmat(p, [1,3]);

Q=repmat(q, [1,3]);

rgb_c= (P + ((Q-P).*6.*t)).*range1 + ...

Q.*range2 + ...

(P + ((Q-P).*6.*(2/3 - t))).*range3 + ...

P.*range4;

rgb_c=round(rgb_c.*10000)./10000;

rgb=reshape(rgb_c, size(hsl_in));

%%%%%%%%%%%%%%%%%%%%%%%%%%%%%%%%%%%%%%%%%%%%%%%%%%%%%%%

function [H, nbHue, hueContrast, missingHue, missingContrast, maxPixel]=hueHistogram(Ihsl,n,C,c)

%[H, nbHue, hueContrast, missingHue, missingContrast,maxPixel]=hueHistogram(Ihsl,n,C,c);

%Constructs of the hue histogram H of the image, counts the number of

%significant hues and missing hues, and calculates their contrasts

%Also returns the percentage of pixels belonging to the most frequent hue

%Construction of the histogram

H=zeros(n,1);

nonConsidered=0;

for i=1:size(Ihsl,1)

for j=1:size(Ihsl,2)

if ((Ihsl(i,j,2)<=0.2) || (Ihsl(i,j,3)>=0.95 || Ihsl(i,j,3)<=0.15))

nonConsidered = nonConsidered+1;

else

for k=1:n

if ((Ihsl(i,j,1)>=(k-1)/n) && (Ihsl(i,j,1)<=k/n))

H(k)=H(k)+1;

end

end

end

end

end

Q=max(H);

nbHue=0;

missingHue=0;

hueContrast=0;

missingContrast=0;

for k=1:n

if (H(k)>(C*Q))

nbHue=nbHue+1;

for l=1:n

if (H(l)>(C*Q))

%contrast is the distance between the two hue

%considered,which are on a wheel

contrast=min(abs((k-l)/n), 1-abs((k-l)/n));

hueContrast=max(hueContrast,contrast);

end

end

end

if (H(k)<(c*Q))

missingHue=missingHue+1;

for l=1:n

if (H(l)<(c*Q))

%contrast is the distance between the two hue

%considered,which are on a wheel

contrast=min(abs((k-l)/n), 1-abs((k-l)/n));

missingContrast=max(missingContrast,contrast);

end

end

end

end

maxPixel=Q/(size(Ihsl,1)*size(Ihsl,2)-nonConsidered);

end

%%%%%%%%%%%%%%%%%%%%%%%%%%%%%%%%%%%%%%%%%%%%%%%%%%%%%%%

function [normalizedDistance]=hueModel(Ih, Is, alpha, k)

% [normalizedDistance]=hueModel(Ih, Is, alpha, k)

% /!\ Ih, Is must be defined and their value must be in [0,1]

% /!\ k must be defined in {1;...;9}

%

% Measure how much the image fits in the k-th hue model, rotated with an

% angle of alpha in [0,360]

colorModel=[180 180 180 180 180;

90 180 270 270 270;

30 65 145 145 145;

80 115 145 145 145;

90 210 240 240 240;

90 90 90 90 90;

30 120 150 240 270;

30 180 210 210 210;

30 30 30 30 30];

IH=mod(Ih*360+alpha,360);

distance=0;

IS=0;

for i=1:size(Ih,1)

for j=1:size(Ih,2)

if ( IH(i,j)<= colorModel(k,1) )

nearestBorder=IH(i,j);

elseif (IH(i,j)>colorModel(k,1))&&(IH(i,j)<colorModel(k,2))

if IH(i,j)-colorModel(k,1)<=colorModel(k,2)-IH(i,j)

nearestBorder=colorModel(k,1);

else

nearestBorder=colorModel(k,2);

end

elseif (IH(i,j)>=colorModel(k,2))&&(IH(i,j)<=colorModel(k,3))

nearestBorder=IH(i,j);

elseif (IH(i,j)>colorModel(k,3))&&(IH(i,j)<colorModel(k,4))

if IH(i,j)-colorModel(k,3)<=colorModel(k,4)-IH(i,j)

nearestBorder=colorModel(k,3);

else

nearestBorder=colorModel(k,4);

end

elseif (IH(i,j)>=colorModel(k,4))&&(IH(i,j)<=colorModel(k,5))

nearestBorder=IH(i,j);

elseif (IH(i,j)>colorModel(k,5))

if IH(i,j)-colorModel(k,5)<=360-IH(i,j)

nearestBorder=colorModel(k,5);

else

nearestBorder=360;

end

end

distance = distance + abs( nearestBorder-IH(i,j) )*Is(i,j);

IS=IS+Is(i,j);

end

end

normalizedDistance=distance/IS;

end

%%%%%%%%%%%%%%%%%%%%%%%%%%%%%%%%%%%%%%%%%%%%%%%%%%%%%%%

function [laplacian_img]=laplacianImage(Ir,Ig,Ib)

% [laplacian_img]=laplacianImage(Ir,Ig,Ib)

% Returns the laplacian image of a picture, Ir Ig and Ib being the red,

% green and blue channels of that picture

% This laplacian image size has been resized to 100x100, and the sum of its

% values has been normalized to 1.

h = fspecial('laplacian', 0.2);

IR=abs(imfilter(Ir,h,'replicate'));

IG=abs(imfilter(Ig,h,'replicate'));

IB=abs(imfilter(Ib,h,'replicate'));

edge_img=(IR+IG+IB)/3;

laplacian_img=imresize(edge_img,[100 100]);

laplacian_img=laplacian_img/norm(laplacian_img(:),1);

end

%%%%%%%%%%%%%%%%%%%%%%%%%%%%%%%%%%%%%%%%%%%%%%%%%%%%%%%

function [M]=laplacianMean(foldername)

% [M]=laplacianMean(foldername)

% Gives the mean across all the 100x100 normalized Laplacian images of the pictures in this folder

%

% /!\ the for loop starts at 3 (should be 4 for mac) because list(1).name='.' and list(2).name='..'

list=dir(foldername);

% /!\ the for loop starts at 3 (should be 4 for mac) because list(1).name='.' and list(2).name='..'

M=zeros(100,100);

for image=3:size(list)

string=strcat(foldername,'/',list(image).name);

Irgb=imread(string);

Irgb=im2double(Irgb);

Ir=Irgb(:,:,1);

Ig=Irgb(:,:,2);

Ib=Irgb(:,:,3);

[laplacian_img]=laplacianImage(Ir,Ig,Ib);

M=M+laplacian_img;

end

size(list,1)

M=M/(size(list,1)-2);

%%%%%%%%%%%%%%%%%%%%%%%%%%%%%%%%%%%%%%%%%%%%%%%%%%%%%%%

function hsl=rgb2hsl(rgb_in)

%Converts Red-Green-Blue Color value to Hue-Saturation-Luminance Color value

%

%Usage

% HSL = rgb2hsl(RGB)

%

% converts RGB, a M [x N] x 3 color matrix with values between 0 and 1

% into HSL, a M [x N] X 3 color matrix with values between 0 and 1

%

%See also hsl2rgb, rgb2hsv, hsv2rgb

% (C) Vladimir Bychkovsky, June 2008

% written using:

% - an implementation by Suresh E Joel, April 26,2003

% - Wikipedia: http://en.wikipedia.org/wiki/HSL_and_HSV

rgb=reshape(rgb_in, [], 3);

mx=max(rgb,[],2);%max of the 3 colors

mn=min(rgb,[],2);%min of the 3 colors

L=(mx+mn)/2;%luminance is half of max value + min value

S=zeros(size(L));

% this set of matrix operations can probably be done as an addition...

zeroidx= (mx==mn);

S(zeroidx)=0;

lowlidx=L <= 0.5;

calc=(mx-mn)./(mx+mn);

idx=lowlidx & (~ zeroidx);

S(idx)=calc(idx);

hilidx=L > 0.5;

calc=(mx-mn)./(2-(mx+mn));

idx=hilidx & (~ zeroidx);

S(idx)=calc(idx);

hsv=rgb2hsv(rgb);

H=hsv(:,1);

hsl=[H, S, L];

hsl=round(hsl.*100000)./100000;

hsl=reshape(hsl, size(rgb_in));

%%%%%%%%%%%%%%%%%%%%%%%%%%%%%%%%%%%%%%%%%%%%%%%%%%%%%%%

% RGB2LUV performs RGB to Luv color conversion.

% LUV = RGB2LUV( RGB ) converts the RGB color vectors into LUV. RGB must

% range in (0.0,1.0).

%

% See also LUV2RGB

%

% 6.891 Problem Set 4: Problem 1

% C. Mario Christoudias

function luv = rgb2luv( rgb )

% convert to XYZ

XYZ = [0.4125, 0.3576, 0.1804; ...

0.2125, 0.7154, 0.0721; ...

0.0193, 0.1192, 0.9502];

xyz = XYZ * rgb;

% convert to Luv

luv = xyz;

Yn = 1;

Lt = 0.008856;

Un_prime = 0.19784977571475;

Vn_prime = 0.46834507665248;

L0 = xyz(2,:) / Yn;

warning off MATLAB:divideByZero;

constant = xyz(1,:) + 15 * xyz(2,:) + 3 * xyz(3,:);

u_prime = (constant ~= 0) .* ((4 * xyz(1,:)) ./ constant) + (constant == 0) * 4.0;

v_prime = (constant ~= 0) .* ((9 * xyz(2,:)) ./ constant) + (constant == 0) * 9.0/15.0;

luv(1,:) = (L0 > Lt) .* (116.0 * (L0 .^ (1/3)) - 16.0) + (L0 <= Lt) .* (903.3 * L0);

luv(2,:) = 13 * luv(1,:) .* (u_prime - Un_prime);

luv(3,:) = 13 * luv(1,:) .* (v_prime - Vn_prime);

% be rid of NaNs

luv(find(isnan(luv))) = 0;

%%%%%%%%%%%%%%%%%%%%%%%%%%%%%%%%%%%%%%%%%%%%%%%%%%%%%%

function [ hist_distance, emd_distance ] = rgbCubes( Ir, Ig, Ib )

%UNTITLED Summary of this function goes here

% Detailed explanation goes here

n=4;

limit=n+1;

n2=n*n;

nbBox=n^3;

IR=n*Ir+1;

IG=n*Ig+1;

IB=n*Ib+1;

distribution=zeros(n,n,n);

for i=1:size(Ir,1)

for j=1:size(Ir,2)

if IR(i,j)==limit

IR(i,j)=n;

end

if IG(i,j)==limit

IG(i,j)=n;

end

if IB(i,j)==limit

IB(i,j)=n;

end

distribution(floor(IR(i,j)),floor(IG(i,j)),floor(IB(i,j)))=distribution(floor(IR(i,j)),floor(IG(i,j)),floor(IB(i,j)))+1;

end

end

H=zeros(nbBox,1);

Y=ones(nbBox,1)/nbBox;

A=zeros(nbBox,nbBox);

D2=zeros(nbBox,nbBox);

max_=0;

for i=1:n

for j=1:n

for k=1:n

H(k+n*(j-1)+n2*(i-1))=distribution(i,j,k);

for i2=1:n

for j2=1:n

for k2=1:n

c1=[0.5+(i-1); 0.5+(j-1); 0.5+(k-1)]/n;

c2=[0.5+(i2-1); 0.5+(j2-1); 0.5+(k2-1)]/n;

C1=rgb2luv(c1);

C2=rgb2luv(c2);

A(k+n*(j-1)+n2*(i-1),k2+n*(j2-1)+n2*(i2-1))=norm(c1-c2,2);

D2(k+n*(j-1)+n2*(i-1),k2+n*(j2-1)+n2*(i2-1))=norm(C1-C2,2);

max_=max(max_,norm(c1-c2,2));

end

end

end

end

end

end

H=H/(size(Ir,1)*size(Ir,2));

A=1-A/max_;

hist_distance=sqrt( transpose(H-Y)*A*(H-Y) );

emd_distance=emd(H,D2,n);

end

%%%%%%%%%%%%%%%%%%%%%%%%%%%%%%%%%%%%%%%%%%%%%%%%%%%%%%%

function luvim = RGBim2Luv(im)

if size(im,3) ~= 3

error('im must have three color channels');

end

if ~isa(im,'float')

im = im2single(im);

end

if (max(im(:)) > 1)

im = im./255;

end

XYZ = [.4125 .3576 .1804; .2125 .7154 .0721; .0193 .1192 .9502];

Yn = 1.0;

Lt = .008856;

Up = 0.19784977571475;

Vp = 0.46834507665248;

imsiz = size(im);

im = permute(im,[3 1 2]);

im = reshape(im,[3 prod(imsiz(1:2))]);

xyz = reshape((XYZ*im)',imsiz);

x = xyz(:,:,1);

y = xyz(:,:,2);

z = xyz(:,:,3);

l0 = y./Yn;

l = l0;

l(l0>Lt) = 116.*(l0(l0>Lt).^(1/3)) - 16;

l(l0<=Lt) = 903.3*l0(l0<=Lt);

c = x + 15*y + 3 * z;

u = 4*ones(imsiz(1:2),class(im));

v = (9/15)*ones(imsiz(1:2),class(im));

u(c~=0) = 4*x(c~=0)./c(c~=0);

v(c~=0) = 9*y(c~=0)./c(c~=0);

u = 13*l.*(u-Up);

v = 13*l.*(v-Vp);

luvim = cat(3,l,u,v);

%%%%%%%%%%%%%%%%%%%%%%%%%%%%%%%%%%%%%%%%%%%%%%%%%%%%%%%

function [nb_cc,avgH,avgS,avgV,XY_100,SI_XY,centroid,color_spread,complem_colors,convexity,centroid_x,centroid_y,shape_variance,shape_skewness,brightness,hue_contrast,saturation_contrast,brightness_contrast,blur_contrast]=seg1(Irgb,k,m)

% [nb_cc,avgH,avgS,avgV,XY_100,SI_XY,centroid,color_spread,complem_colors,...

% convexity,centroid_x,centroid_y,shape_variance,shape_skewness,brightness,...

% hue_contrast,saturation_contrast,brightness_contrast,blur_contrast]=seg1(Irgb,k,m)

%

% Performs a color-based segmentation process on our image in the LUV color

% space, using kmeans and bnconncomp

% Then, returns all the segmentation related features (in matrices)

%

% Returns an error if the image contains less than 5 'objects'

Ir=Irgb(:,:,1);

Ig=Irgb(:,:,2);

Ib=Irgb(:,:,3);

Ihsv=rgb2hsv(Irgb);

Ih=Ihsv(:,:,1);

Is=Ihsv(:,:,2);

Iv=Ihsv(:,:,3);

Iluv=RGBim2Luv(Irgb);

I(:,:,1)=context(Iluv(:,:,1),m);

I(:,:,2)=context(Iluv(:,:,2),m);

I(:,:,3)=context(Iluv(:,:,3),m);

%we have to reshape Iluv to apply kmeans to it

X=size(I,1);

Y=size(I,2);

XY=X*Y;

iLuv=reshape(I,[XY 3]);

ID=kmeans(iLuv,k);

%we now have an image where each pixel has an indice i in [1,k],

%corresponding to the cluster it is in

cluster=reshape(ID,[X Y]);

imtool(cluster);

%we now want to find patches of connected components

binary_cluster=cell(1,k);

connected_cluster=cell(1,k);

num_pixels=cell(1,k);

k_biggest=zeros(2,k);

nb_cc=0;

for j=1:k

binary_cluster{j}=cluster<j+1;

cluster=cluster+k*binary_cluster{j};

connected_cluster{j}=bwconncomp(binary_cluster{j});

nb_cc=nb_cc+connected_cluster{j}.NumObjects;

num_pixels{j}=cellfun(@numel,connected_cluster{j}.PixelIdxList);

[k_biggest(1,j),k_biggest(2,j)]=max(num_pixels{j}); %biggest(1,k)=nb of pixel in it, biggest(1,k)= its index number

end

%we want to select the 5-th biggest connected components and

%analyse them

if nb_cc<5

error('/!\ ERROR: This image analysis produce less than 5 connected components! It means that that we can"t analyse its 5 biggest connected components, and that some results ARE false.');

end

biggest_patches=cell(2,5); %1st row for the number of pixels in the patch, 2nd row for the list of the indices of the pixels in this patch

XY_100=0;

avgH=-1*ones(5,1);

avgS=-1*ones(5,1);

avgV=-1*ones(5,1);

SI_XY=zeros(5,1);

centroid=zeros(5,1);

lightness=zeros(5,1);

num_pixels_=num_pixels; %we need to modifie num_pixels in the loop, but we will also need it later

centroid_x=zeros(3,1);

centroid_y=zeros(3,1);

shape_variance=zeros(3,1);

shape_skewness=zeros(3,1);

brightness=zeros(3,1);

blur_matrix=zeros(size(Ir));

blur=zeros(5,1);

for i=1:5;

%finds the new biggest patch and puts its information in biggest_patches

[biggest_patches{1,i},J]=max(k_biggest(1,:)); %J is the index of the cluster this patch is from (in [1,k])

IDX=k_biggest(2,J); %IDX is its index in connected_cluster{J}.PixelIdxList

biggest_patches{2,i}=connected_cluster{J}.PixelIdxList{IDX};

%construction of the features linked to the i-th patch

if biggest_patches{1,i}>XY/100

XY_100=XY_100+1;

end

avgH(i)=mean(Ih(biggest_patches{2,i}));

avgS(i)=mean(Is(biggest_patches{2,i}));

avgV(i)=mean(Iv(biggest_patches{2,i}));

SI_XY(i)=biggest_patches{1,i}/XY;

avg_x=mean(mod(biggest_patches{2,i}-1,X)+1);

avg_y=mean(floor((biggest_patches{2,i}-1)/X)+1);

r=floor(3*avg_x/X)+1;

c=floor(3*avg_y/Y)+1;

centroid(i)=10*r+c;

lightness(i)=mean(Ir(biggest_patches{2,i})+Ig(biggest_patches{2,i})+Ib(biggest_patches{2,i}));

blur_matrix(biggest_patches{2,i})=(Ir(biggest_patches{2,i})+Ig(biggest_patches{2,i})+Ib(biggest_patches{2,i}))/3;

blur(i)=gaussian_blur(blur_matrix,blur_matrix,blur_matrix);

num_pixels_{J}(IDX)=0;

[k_biggest(1,J),k_biggest(2,J)]=max(num_pixels_{J});

%those features from paper2 only consider the 3 biggest segments

if i<4

centroid_x(i)=avg_x/X;

centroid_y(i)=avg_y/Y;

difference_x=((mod(biggest_patches{2,i}-1,X)+1)-avg_x)/X;

difference_y=((floor((biggest_patches{2,i}-1)/X)+1)-avg_y)/Y;

for j=1:biggest_patches{1,i}

shape_variance(i)=shape_variance(i)+difference_x(j)^2+difference_y(j)^2;

shape_skewness(i)=shape_skewness(i)+difference_x(j)^3+difference_y(j)^3;

end

shape_variance(i)=shape_variance(i)/biggest_patches{1,i};

shape_skewness(i)=shape_skewness(i)/biggest_patches{1,i};

brightness(i)=lightness(i);

end

end

%features from paper1

color_spread=0;

complem_colors=0;

%features from paper2

hue_contrast=0;

saturation_contrast=0;

brightness_contrast=0;

blur_contrast=0;

for i=1:5

for j=1:5

color_spread=color_spread+abs(avgH(i)-avgH(j));

complem_colors=complem_colors+min(abs(avgH(i)-avgH(j)),1-abs(avgH(i)-avgH(j)));

hue_contrast=max(hue_contrast,min(abs(avgH(i)-avgH(j)),1-abs(avgH(i)-avgH(j))));

saturation_contrast=max(saturation_contrast,abs(avgS(i)-avgS(j)));

brightness_contrast=max(brightness_contrast,abs(lightness(i)-lightness(j)));

blur_contrast=max(blur_contrast,abs(blur(i)-blur(j)));

end

end

%analysis of the convexity of the shapes in the image

convexity=0;

for j=1:k

%we first need to detect all the shapes which surface is >200/XY

for i=1:size(num_pixels{j},2)

if (num_pixels{j}(i))>(XY/200)

pixel_list=cell2mat(connected_cluster{j}.PixelIdxList(i));

shape_x=mod(pixel_list-1,X)+1;

shape_y=floor((pixel_list-1)/X)+1;

[~,conv_area]=convhull(shape_x,shape_y);

if (num_pixels{j}(i)/conv_area)>0.8

convexity=convexity+num_pixels{j}(i);

end

end

end

end

convexity=convexity/XY;

end

%%%%%%%%%%%%%%%%%%%%%%%%%%%%%%%%%%%%%%%%%%%%%%%%%%%%%%%

function [ a ] = thirdsAvg( M,margin )

% [ a ] = thirdsAvg( M,margin )

% Returns the average 'a' of the central part matrix with respect to rule of thirds

% This central part is defined as the central ninth of the photo if you

% divide it along the 1/3 and the 2/3 of its height and width, with a

% margin of 'margin' percent around it.

%

%/!\margin must be in [0,1]

v=0;

for x=floor((1-margin)*size(M,1)/3):floor((1+margin)*(2*size(M,1))/3)

for y=floor((1-margin)*size(M,2)/3):floor((1+margin)*(2*size(M,2))/3)

v=v+M(x,y);

end

end

a=v/((floor((1+margin)*(2*size(M,1))/3)-floor((1-margin)*size(M,1)/3))*(floor((1+margin)*(2*size(M,2))/3)-floor((1-margin)*size(M,2)/3)));

end

%%%%%%%%%%%%%%%%%%%%%%%%%%%%%%%%%%%%%%%%%%%%%%%%%%%%%%%

function [cA,cH,cV,cD]=wavelet(I)

% [cA,cH,cV,cD]=wavelet(I)

% Performs a three-level Haar wavelet transform on I, and returns the cells

% cA, cH, cV and cD where forall i in {1,2,3}, cA{i} is the i-th level approximation

% coefficients matrix, and cH{i}, cV{i} and cD{i} are the i-th level

% details coefficients matrices

cA=cell(1,3);

cH=cell(1,3);

cV=cell(1,3);

cD=cell(1,3);

%Performing 3 level Daubechies tranform on I

startImage = I;

for i = 1:3,

[cA{i},cH{i},cV{i},cD{i}] = dwt2(startImage,'haar');

startImage = cA{i};

end

end

%%%%%%%%%%%%%%%%%%%%%%%%%%%%%%%%%%%%%%%%%%%%%%%%%%%%%%%

function [texture,low_DOF]=waveletTexture(Ihsv)

% [texture,low_DOF]=waveletTexture(Ihsv)

% Returns the 4x3 matrix 'texture' containing 12 wavelet features to

% analyse the texture of the image (cf paper1), and a 3x1 matrix 'low_DOF'

% containing a measure of the low depth of field of the image.

%

% In 'texture': lines 1,2,3,4 correspond to the 1st, 2nd and 3rd level and

% to the sum of the average wavelet coefficients of the column, and columns 1,2,3 to h,s,v

% In 'low_DOF': the 1st row contains the low DOF feature for H, the 2nd row the one for S, and the 3rd row the one for V

X_4=floor(size(Ihsv,1)/(8*4));

X3_4=floor(3*size(Ihsv,1)/(8*4));

Y_4=floor(size(Ihsv,2)/(8*4));

Y3_4=floor(3*size(Ihsv,2)/(8*4));

%applies a 3-level wavelet transform

[cA,cH,cV,cD]=wavelet(Ihsv);

%The matrix containing the results (lines 1,2,3,4 correspond to the 1st, 2nd and 3rd level and to the sum of the average wavelet coefficients of the column, columns 1,2,3 to h,s,v)

texture=zeros(4,3);

low_DOF=zeros(3,1); %1st row for H, 2nd for S, 3rd for V

sH=zeros(3,1);

sS=zeros(3,1);

sV=zeros(3,1);

%construction of the k-level features

for k = 1:3

sumH=cH{k}(:,:,1) + cV{k}(:,:,1) + cD{k}(:,:,1);

sumS=cH{k}(:,:,2) + cV{k}(:,:,2) + cD{k}(:,:,2);

sumV=cH{k}(:,:,3) + cV{k}(:,:,3) + cD{k}(:,:,3);

%L1 norm

absH=abs(cH{k}(:,:,1)) + abs(cV{k}(:,:,1)) + abs(cD{k}(:,:,1));

absS=abs(cH{k}(:,:,2)) + abs(cV{k}(:,:,2)) + abs(cD{k}(:,:,2));

absV=abs(cH{k}(:,:,3)) + abs(cV{k}(:,:,3)) + abs(cD{k}(:,:,3));

sH(k,1)=norm(absH(:),1);

sS(k,1)=norm(absS(:),1);

sV(k,1)=norm(absV(:),1);

for i = 1:floor(size(Ihsv,1)/(2^k))

for j = 1:floor(size(Ihsv,2)/(2^k))

texture(k,1)=texture(k,1) + sumH(i,j);

texture(k,2)=texture(k,2) + sumS(i,j);

texture(k,3)=texture(k,3) + sumV(i,j);

if ((k==3)&&(((i>X_4)&&(i<X3_4))&&((j>Y_4)&&(j<Y3_4))))

low_DOF(1)=low_DOF(1)+ sumH(i,j);

low_DOF(2)=low_DOF(2)+ sumS(i,j);

low_DOF(3)=low_DOF(3)+ sumV(i,j);

end

end

end

if k==3

low_DOF(1)=low_DOF(1)/texture(k,1);

low_DOF(2)=low_DOF(2)/texture(k,2);

low_DOF(3)=low_DOF(3)/texture(k,3);

end

texture(k,1)=texture(k,1)/sH(k,1);

texture(k,2)=texture(k,2)/sS(k,1);

texture(k,3)=texture(k,3)/sV(k,1);

end

texture(4,1)=texture(1,1)+texture(2,1)+texture(3,1);

texture(4,2)=texture(1,2)+texture(2,2)+texture(3,2);

texture(4,3)=texture(1,3)+texture(2,3)+texture(3,3);

end
