# Supplementary material for: Can we measure beauty? Computational evaluation of coral reef aesthetics
Source: PeerJ. 2015 Nov 10;3:e1390. doi: 10.7717/peerj.1390 (PMC4647610; doi:10.7717/peerj.1390)
Supplement: Table S1 — Overview of all 109 implemented features given along their relative importance for the combined coral reef aesthetic value, a short description and the study the respective feature was derived from (1, Datta et al., 2006; 2, Li & Chen, 2009; 3, Ke, Tang & Jing, 2006; 4, this study). [file peerj-03-1390-s004.docx]

| **Feature** | **Relative importance** | **Computational implementation** | **Description** | **Feature group** | **Reference** |
| --- | --- | --- | --- | --- | --- |
| *f_1_* | 0.724 | $\frac{1}{MN} \sum_{n} \sum_{m}$*I_H_(m, n)* | Average hue across the whole image (in HSV color space) | Color | 1, 2 |
| *f_2_* | 0.456 | $\frac{1}{MN} \sum_{n} \sum_{m}$*I_S_(m, n)* | Average saturation across the whole image (in HSV color space) | Color | 1 |
| *f_3_* | 0.876 | $\frac{1}{MN} \sum_{n} \sum_{m}$*I_V_(m, n)* | Average value across the whole image (in HSV color space) | Color | 1 |
| *f_4_* | 0.592 | $\frac{1}{MN} \sum_{n} \sum_{m}$*I_S__(m, n)* | Average saturation across the whole image (in HSL color space) | Color | 2 |
| *f_5_* | 0.733 | $\frac{1}{MN} \sum_{n} \sum_{m}$*I_L__(m, n)* | Average brightness across the whole image (in HSL color space) | Color | 2 |
| *f_6_* | 0.338 | $\sqrt{\left( h-h\text{0} \right)\text{T }A\left( h-h\text{0} \right)}$ | Colorfulness using quadratic-form distance | Color | 1 |
| *f_7_* | 0.455 | *emd(D_1_, D_0_, {d_ij_\|1 < i,j < 64})* | Colorfulness using earth mover’s distance | Color | 1 |
| *f_8_* | 0.761 | *min(h_max_)* | Value of most frequent hue | Color | 4 |
| *f_9_* | 0.614 | *std(var(I’_H__))* | Standard deviation of colorfulness | Color | 4 |
| *f_10_* | 0.285 | *# of {i\|h(i) > C • Q}* | Number of hues present | Color | 2 |
| *f_11_* | 0.470 | *# of {i\|h(i) > c • Q}* | Number of hues missing | Color | 2, 4 |
| *f_12_* | 0.270 | *max(\|\|c_h_(i) – c_h_(j)\|\|_al_) with i, j ∈ {i\|h(i) > C • Q}* | Hue contrast across image | Color | 2 |
| *f_13_* | 0.357 | *max(\|\|c_h_(i) – c_h_(j)\|\|_al_) with i, j ∈ {i\|h(i) < c • Q}* | Contrast between missing hues across image | Color | 2, 4 |
| *f_14_* | 0.451 | *= Q/N where N = # of P_H_* | Number of pixels belonging to most frequent hue | Color | 2 |
| *f_15_* | 0.357 | *20 – # of {i\|h(i) > C_2_ • Q} with C_2_ = 0.05* | Number of significant hues in image | Color | 23 |
| *f_16_* | 0.554 | $\min_{\alpha}$ *F_1,α_,* | Distance to 1^st^ hue model | Color | 2, 4 |
| *f_17_* | 0.463 | $\min_{\alpha}$ *F_2,α_,* | Distance to 2^nd^ hue model | Color | 2, 4 |
| *f_18_* | 0.523 | $\min_{\alpha}$ *F_3,α_,* | Distance to 3^rd^ hue model | Color | 2, 4 |
| *f_19_* | 0.554 | $\min_{\alpha}$ *F_4,α_,* | Distance to 4^th^ hue model | Color | 2, 4 |
| *f_20_* | 0.521 | $\min_{\alpha}$ *F_5,α_,* | Distance to 5^th^ hue model | Color | 2, 4 |
| *f_21_* | 0.509 | $\min_{\alpha}$ *F_6,α_,* | Distance to 6^th^ hue model | Color | 2, 4 |
| *f_22_* | 0.528 | $\min_{\alpha}$ *F_7,α_,* | Distance to 7^th^ hue model | Color | 2, 4 |
| *f_23_* | 0.495 | $\min_{\alpha}$ *F_8,α_,* | Distance to 8^th^ hue model | Color | 2, 4 |
| *f_24_* | 0.519 | $\min_{\alpha}$ *F_9,α_,* | Distance to 9^th^ hue model | Color | 2, 4 |
| *f_25_* | 0.199 | $\left\{ \begin{aligned} \text{max}\text{k}\text{∈}\text{\{j\vert Fj,α(j),TH\}}\text{ k if ∃k}\text{∈}\text{ \{1, …, 9\}, F}\text{k,α(k)}\text{ < TH} \\ \text{k}\text{0 }\text{if }\text{∀}\text{k F}\text{k,α(k)}\text{ ≥ TH} \end{aligned} \right\}$ | Best fitting hue model | Color | 2 |
| *f_26_* | 0.809 | $\frac{1}{MN} \sum_{m} \sum_{n}$*L(m, n)* | Arithmetic average of brightness | Color | 2, 3 |
| *f_27_* | 0.800 | *exp(* $\frac{255}{MN} \sum_{m} \sum_{n}$*log(_∈_ +* $\frac{L(m, n)}{255}$*))* | Logarithmic average of brightness | Color | 2 |
| *f_28_* | 0.444 | *b - a + 1* | Brightness contrast across image (100 bin histogram) | Color | 2 |
| *f_29_* | 0.708 | *b_2_ – a_2_* | Brightness contrast across image (255 bin histogram) | Color | 3 |
| *f_30_* | 0.901 | *H_90_W_90_/HW* | Area of bounding box containing 81% of edge energy | Texture | 2 |
| *f_31_* | 0.922 | *1 – H_98_W_98_; H_98_ and W_98_ ∈ [0, 1]* | Resized area of bounding box containing 96.04% of edge energy | Texture | 23 |
| *f_32_* | 1.165 | *d_s_ – d_p_* | Similarity measure of high frequency edges spatial distribution | Texture | 23 |
| *f_33_* | 0.739 | *(\|S_r_\|_L1_ + \|S_g_\|_L1_1 + \|S_b_\|_L1_1)/3* | Sum of edges | Texture | 4 |
| *f_34_* | 0.814 | $\frac{1}{MN} \sum_{m} \sum_{n}$*(R_H_(m, n) + R_S_(m, n) + R_V_(m, n))/3* | Range of texture | Texture | 4 |
| *f_35_* | 0.767 | $\frac{1}{MN} \sum_{m} \sum_{n}$*(D_H_(m, n) + D_S_(m, n) + D_V_(m, n))/3* | Average standard deviation of texture | Texture | 4 |
| *f_36_* | 0.476 | *entropy(I_r_)* | Entropy of red matrix I_r_ | Texture | 4 |
| *f_37_* | 0.630 | *entropy(I_g_)* | Entropy of green matrix I_g_ | Texture | 4 |
| *f_38_* | 0.862 | *entropy(I_b_)* | Entropy of blue matrix I_b_ | Texture | 4 |
| *f_39_* | 1.138 | $\frac{1}{\vert C{}_{1}^{H}{}\vert L1} \sum_{m} \sum_{n}$*(*$C_{1}^{H}$*(m, n) +* $C_{1}^{V}$ *(m, n) +* $C_{1}^{D}$*(m, n))* | Wavelet feature level 1 for H | Texture | 1 |
| *f_40_* | 0.744 | $\frac{1}{\vert C{}_{2}^{H}{}\vert L1} \sum_{m} \sum_{n}$*(*$C_{2}^{H}$*(m, n) +* $C_{2}^{V}$ *(m, n) +* $C_{2}^{D}$*(m, n))* | Wavelet feature level 2 for H | Texture | 1 |
| *f_41_* | 0.957 | $\frac{1}{\vert C{}_{3}^{H}{}\vert L1} \sum_{m} \sum_{n}$*(*$C_{3}^{H}$*(m, n) +* $C_{3}^{V}$ *(m, n) +* $C_{3}^{D}$*(m, n))* | Wavelet feature level 3 for H | Texture | 1 |
| *f_42_* | 0.892 | $\frac{1}{\vert C{}_{1}^{S}{}\vert L1} \sum_{m} \sum_{n}$*(*$C_{1}^{H}$*(m, n) +* $C_{1}^{V}$ *(m, n) +* $C_{1}^{D}$*(m, n))* | Wavelet feature level 1 for S | Texture | 1 |
| *f_43_* | 0.600 | $\frac{1}{\vert C{}_{2}^{S}{}\vert L1} \sum_{m} \sum_{n}$*(*$C_{2}^{H}$*(m, n) +* $C_{2}^{V}$ *(m, n) +* $C_{2}^{D}$*(m, n))* | Wavelet feature level 2 for S | Texture | 1 |
| *f_44_* | 0.626 | $\frac{1}{\vert C{}_{3}^{S}{}\vert L1} \sum_{m} \sum_{n}$*(*$C_{3}^{H}$*(m, n) +* $C_{3}^{V}$ *(m, n) +* $C_{3}^{D}$*(m, n))* | Wavelet feature level 3 for S | Texture | 1 |
| *f_45_* | 1.060 | $\frac{1}{\vert C{}_{1}^{V}{}\vert L1} \sum_{m} \sum_{n}$*(*$C_{1}^{H}$*(m, n) +* $C_{1}^{V}$ *(m, n) +* $C_{1}^{D}$*(m, n))* | Wavelet feature level 1 for V | Texture | 1 |
| *f_46_* | 0.932 | $\frac{1}{\vert C{}_{2}^{V}{}\vert L1} \sum_{m} \sum_{n}$*(*$C_{2}^{H}$*(m, n) +* $C_{2}^{V}$ *(m, n) +* $C_{2}^{D}$*(m, n))* | Wavelet feature level 2 for V | Texture | 1 |
| *f_47_* | 0.590 | $\frac{1}{\vert C{}_{3}^{V}{}\vert L1} \sum_{m} \sum_{n}$*(*$C_{3}^{H}$*(m, n) +* $C_{3}^{V}$ *(m, n) +* $C_{3}^{D}$*(m, n))* | Wavelet feature level 3 for V | Texture | 1 |
| *f_48_* | 1.073 | $\sum_{i=40}^{42}$*f_i,_* | Wavelet feature level 1 (avg) | Texture | 1 |
| *f_49_* | 0.704 | $\sum_{i=43}^{45}$*f_i,_* | Wavelet feature level 2 (avg) | Texture | 1 |
| *f_50_* | 0.832 | $\sum_{i=46}^{48}$*f_i_* | Wavelet feature level 3 (avg) | Texture | 1 |
| *f_51_* | 0.730 | *max* $\left( 2\frac{m^{'}- \left[ \frac{M}{2} \right]}{M};2\frac{n^{'}- \left[ \frac{N}{2} \right]}{N} \right)$ | Blur measure | Texture | 2, 3 |
| *f_52_* | 0.640 | $\frac{1}{\left( \left[ \frac{2M}{3} \right]- \left[ \frac{M}{3} \right]+1 \right)\left( \left[ \frac{2N}{3} \right]- \left[ \frac{N}{3} \right]+1 \right)}$ $\sum_{m= \left[ \frac{M}{3} \right]}^{\left[ \frac{2M}{3} \right]} \sum_{n= \left[ \frac{N}{3} \right]}^{\left[ \frac{2N}{3} \right]}$*I_H_(m, n)* | Average hue (rule of thirds, HSV) | Color | 1 |
| *f_53_* | 0.367 | $\frac{1}{\left( \left[ \frac{2M}{3} \right]- \left[ \frac{M}{3} \right]+1 \right)\left( \left[ \frac{2N}{3} \right]- \left[ \frac{N}{3} \right]+1 \right)}$ $\sum_{m= \left[ \frac{M}{3} \right]}^{\left[ \frac{2M}{3} \right]} \sum_{n= \left[ \frac{N}{3} \right]}^{\left[ \frac{2N}{3} \right]}$*I_S_(m, n)* | Average saturation (rule of thirds, HSV) | Color | 1 |
| *f_54_* | 0.689 | $\frac{1}{\left( \left[ \frac{2M}{3} \right]- \left[ \frac{M}{3} \right]+1 \right)\left( \left[ \frac{2N}{3} \right]- \left[ \frac{N}{3} \right]+1 \right)}$ $\sum_{m= \left[ \frac{M}{3} \right]}^{\left[ \frac{2M}{3} \right]} \sum_{n= \left[ \frac{N}{3} \right]}^{\left[ \frac{2N}{3} \right]}$*I_V_(m, n)* | Average value (rule of thirds, HSV) | Color | 1 |
| *f_55_* | 0.711 | $\frac{1}{\# of \{(m, n)\vert\left( m, n \right)\in FR\}}\sum_{\left( m, n \right)\in FR}$*I_H__(m, n)* | Average hue for focus region (HSL) | Color | 2 |
| *f_56_* | 0.496 | $\frac{1}{\# of \{(m, n)\vert\left( m, n \right)\in FR\}}\sum_{\left( m, n \right)\in FR}$*I_S__(m, n)* | Average saturation for focus region (HSL) | Color | 2 |
| *f_57_* | 0.771 | $\frac{1}{\# of \{(m, n)\vert\left( m, n \right)\in FR\}}\sum_{\left( m, n \right)\in FR}$*I_L__(m, n)* | Average brightness for focus region (HSL) | Color | 2 |
| *f_58_* | 1.066 | # of *L* | Number of color based clusters formed by K-Means (LUV) | Objects | 1 |
| *f_59_* | 0.367 | *# of {s_i_\|# of s_i_ > MN/100}* | Number of segments *s_i_* larger than 1% of the image (*i* in [1,5]) | Objects | 1 |
| *f_60_* | 0.488 | *(# of s_1_)/MN* | Ration of size of largest segment to size of whole image | Objects | 1 |
| *f_61_* | 0.486 | *(# of s_2_)/MN* | Ration of size of 2^nd^ largest segment to size of whole image | Objects | 1 |
| *f_62_* | 0.454 | *(# of s_3_)/MN* | Ration of size of 3^rd^ largest segment to size of whole image | Objects | 1 |
| *f_63_* | 0.549 | *(# of s_4_)/MN* | Ration of size of 4^th^ largest segment to size of whole image | Objects | 1 |
| *f_64_* | 0.539 | *(# of s_5_)/MN* | Ration of size of 5^th^ largest segment to size of whole image | Objects | 1 |
| *f_65_* | 0.217 | *10 * r + c, ∀∈ {1}* | Block containing centroid of 1^st^ cluster | Objects | 1 |
| *f_66_* | 0.136 | *10 * r + c, ∀∈ {2}* | Block containing centroid of 2^nd^ cluster | Objects | 1 |
| *f_67_* | 0.097 | *10 * r + c, ∀∈ {3}* | Block containing centroid of 3^rd^ cluster | Objects | 1 |
| *f_68_* | 0.013 | *10 * r + c, ∀∈ {4}* | Block containing centroid of 4^th^ cluster | Objects | 1 |
| *f_69_* | 0.059 | *10 * r + c, ∀∈ {5}* | Block containing centroid of 5^th^ cluster | Objects | 1 |
| *f_70_* | 0.605 | $\frac{\text{1}}{\text{\# of s}\text{1}\text{ }}$ $\sum_{\left( m, n \right)\in\text{ s}\text{1}\text{ }}$*I_H_(m, n)* | Average hue of largest segment (HSV) | Objects (Color) | 1 |
| *f_71_* | 0.603 | $\frac{\text{1}}{\text{\# of s}\text{2}\text{ }}$ $\sum_{\left( m, n \right)\in\text{ s}\text{2}\text{ }}$*I_H_(m, n)* | Average hue of 2^nd^ largest segment (HSV) | Objects (Color) | 1, 2 |
| *f_72_* | 0.545 | $\frac{\text{1}}{\text{\# of s}\text{3}\text{ }}$ $\sum_{\left( m, n \right)\in\text{ s}\text{3}\text{ }}$*I_H_(m, n)* | Average hue of 3^rd^ largest segment (HSV) | Objects (Color) | 1, 2 |
| *f_73_* | 0.463 | $\frac{\text{1}}{\text{\# of s}\text{4}\text{ }}$ $\sum_{\left( m, n \right)\in\text{ s}\text{4}\text{ }}$*I_H_(m, n)* | Average hue of 4^th^ largest segment (HSV) | Objects (Color) | 1 |
| *f_74_* | 0.506 | $\frac{\text{1}}{\text{\# of s}\text{5}\text{ }}$ $\sum_{\left( m, n \right)\in\text{ s}\text{5}\text{ }}$*I_H_(m, n)* | Average hue of 5^th^ largest segment (HSV) | Objects (Color) | 1 |
| *f_75_* | 0.429 | $\frac{\text{1}}{\text{\# of s}\text{1}\text{ }}$ $\sum_{\left( m, n \right)\in\text{ s}\text{1}\text{ }}$*I_S_(m, n)* | Average saturation of largest segment (HSV) | Objects (Color) | 1, 2 |
| *f_76_* | 0.459 | $\frac{\text{1}}{\text{\# of s}\text{2}\text{ }}$ $\sum_{\left( m, n \right)\in\text{ s}\text{2}\text{ }}$*I_S_(m, n)* | Average saturation of 2^nd^ largest segment (HSV) | Objects (Color) | 1, 2 |
| *f_77_* | 0.357 | $\frac{\text{1}}{\text{\# of s}\text{3}\text{ }}$ $\sum_{\left( m, n \right)\in\text{ s}\text{3}\text{ }}$*I_S_(m, n)* | Average saturation of 3^rd^ largest segment (HSV) | Objects (Color) | 1, 2 |
| *f_78_* | 0.396 | $\frac{\text{1}}{\text{\# of s}\text{4}\text{ }}$ $\sum_{\left( m, n \right)\in\text{ s}\text{4}\text{ }}$*I_S_(m, n)* | Average saturation of 4^th^ largest segment (HSV) | Objects (Color) | 1 |
| *f_79_* | 0.316 | $\frac{\text{1}}{\text{\# of s}\text{5}\text{ }}$ $\sum_{\left( m, n \right)\in\text{ s}\text{5}\text{ }}$*I_S_(m, n)* | Average saturation of 5^th^ largest segment (HSV) | Objects (Color) | 1 |
| *f_80_* | 0.682 | $\frac{\text{1}}{\text{\# of s}\text{1}\text{ }}$ $\sum_{\left( m, n \right)\in\text{ s}\text{1}\text{ }}$*I_V_(m, n)* | Average value of largest segment (HSV) | Objects (Color) | 1 |
| *f_81_* | 0.471 | $\frac{\text{1}}{\text{\# of s}\text{2}\text{ }}$ $\sum_{\left( m, n \right)\in\text{ s}\text{2}\text{ }}$*I_V_(m, n)* | Average value of 2^nd^ largest segment (HSV) | Objects (Color) | 1 |
| *f_82_* | 0.378 | $\frac{\text{1}}{\text{\# of s}\text{3}\text{ }}$ $\sum_{\left( m, n \right)\in\text{ s}\text{3}\text{ }}$*I_V_(m, n)* | Average value of 3^rd^ largest segment (HSV) | Objects (Color) | 1 |
| *f_83_* | 0.343 | $\frac{\text{1}}{\text{\# of s}\text{4}\text{ }}$ $\sum_{\left( m, n \right)\in\text{ s}\text{4}\text{ }}$*I_V_(m, n)* | Average value of 4^th^ largest segment (HSV) | Objects (Color) | 1 |
| *f_84_* | 0.321 | $\frac{\text{1}}{\text{\# of s}\text{5}\text{ }}$ $\sum_{\left( m, n \right)\in\text{ s}\text{5}\text{ }}$*I_V_(m, n)* | Average value of 5^th^ largest segment (HSV) | Objects (Color) | 1 |
| *f_85_* | 0.544 | $\frac{\text{1}}{\text{\# of s}\text{1}\text{ }}$ $\sum_{\left( m, n \right)\in\text{ s}\text{1}\text{ }}$*L(m, n)* | Average brightness of largest segment (HSV) | Objects (Color) | 2 |
| *f_86_* | 0.411 | $\frac{\text{1}}{\text{\# of s}\text{2}\text{ }}$ $\sum_{\left( m, n \right)\in\text{ s}\text{2}\text{ }}$*L(m, n)* | Average brightness of 2^nd^ largest segment (HSV) | Objects (Color) | 2 |
| *f_87_* | 0.260 | $\frac{\text{1}}{\text{\# of s}\text{3}\text{ }}$ $\sum_{\left( m, n \right)\in\text{ s}\text{3}\text{ }}$*L(m, n)* | Average brightness of 3^rd^ largest segment (HSV) | Objects (Color) | 2 |
| *f_88_* | 0.334 | $\sum_{i=1}^{5} \sum_{j=1}^{5}$*\|h(i) – h(j)\|* | Average color spread among top 5 patch hues | Color | 1 |
| *f_89_* | 0.354 | $\sum_{i=1}^{5} \sum_{j=1}^{5}$*\|\|h(i) – h(j)\|\|_al_* | Average complimentary colors among top 5 patch hues | Color | 1 |
| *f_90_* | 0.367 | *x_1_ =* $\frac{\text{1}}{\text{\# of s}\text{1}\text{ }}$ $\sum_{\left( m, n \right)\in\text{ s}\text{1}\text{ }}$*x(m, n)* | Horizontal coordinate of mass center for largest segment | Objects | 2 |
| *f_91_* | 0.190 | *x_2_ =* $\frac{\text{1}}{\text{\# of s}\text{2}\text{ }}$ $\sum_{\left( m, n \right)\in\text{ s}\text{2}\text{ }}$*x(m, n)* | Horizontal coordinate of mass center for 2^nd^ largest segment | Objects | 2 |
| *f_92_* | 0.135 | *x_3_ =* $\frac{\text{1}}{\text{\# of s}\text{3}\text{ }}$ $\sum_{\left( m, n \right)\in\text{ s}\text{3}\text{ }}$*x(m, n)* | Horizontal coordinate of mass center for 3^rd^ largest segment | Objects | 2 |
| *f_93_* | 0.133 | *͞y_1_ =* $\frac{\text{1}}{\text{\# of s}\text{1}\text{ }}$ $\sum_{\left( m, n \right)\in\text{ s}\text{1}\text{ }}$*y(m, n)* | Vertical coordinate of mass center for largest segment | Objects | 2 |
| *f_94_* | 0.096 | *͞y_2_ =* $\frac{\text{1}}{\text{\# of s}\text{2}\text{ }}$ $\sum_{\left( m, n \right)\in\text{ s}\text{2}\text{ }}$*y(m, n)* | Vertical coordinate of mass center for 2^nd^ largest segment | Objects | 2 |
| *f_95_* | 0.091 | *͞y_3_ =* $\frac{\text{1}}{\text{\# of s}\text{3}\text{ }}$ $\sum_{\left( m, n \right)\in\text{ s}\text{3}\text{ }}$*y(m, n)* | Vertical coordinate of mass center for 3^rd^ largest segment | Objects | 2 |
| *f_96_* | 0.418 | $\frac{\text{1}}{\text{\# of s}\text{1}\text{ }}$ $\sum_{\left( m, n \right)\in\text{ s}\text{1}\text{ }}$*((x(m, n) – ͞x_1_ )^2^ + ((y(m, n) – ͞y_1_ )^2^)* | Mass variance for largest segment | Objects | 2 |
| *f_97_* | 0.346 | $\frac{\text{1}}{\text{\# of s}\text{2}\text{ }}$ $\sum_{\left( m, n \right)\in\text{ s}\text{2}\text{ }}$*((x(m, n) – ͞x_2_ )^2^ + ((y(m, n) – ͞y_2_ )^2^)* | Mass variance for 2^nd^ largest segment | Objects | 2 |
| *f_98_* | 0.421 | $\frac{\text{1}}{\text{\# of s}\text{3}\text{ }}$ $\sum_{\left( m, n \right)\in\text{ s}\text{3}\text{ }}$*((x(m, n) – ͞x_3_ )^2^ + ((y(m, n) – ͞y_3_ )^2^)* | Mass variance for 3^rd^ largest segment | Objects | 2 |
| *f_99_* | 0.062 | $\frac{\text{1}}{\text{\# of s}\text{1}\text{ }}$ $\sum_{\left( m, n \right)\in\text{ s}\text{1}\text{ }}$*((x(m, n) – ͞x_1_ )^3^ + ((y(m, n) – ͞y_1_ )^3^)* | Mass skewness for largest segment | Objects | 2 |
| *f_100_* | 0.152 | $\frac{\text{1}}{\text{\# of s}\text{2}\text{ }}$ $\sum_{\left( m, n \right)\in\text{ s}\text{2}\text{ }}$*((x(m, n) – ͞x_2_ )^3^ + ((y(m, n) – ͞y_2_ )^3^)* | Mass skewness for 2^nd^ largest segment | Objects | 2 |
| *f_101_* | 0.109 | $\frac{\text{1}}{\text{\# of s}\text{3}\text{ }}$ $\sum_{\left( m, n \right)\in\text{ s}\text{3}\text{ }}$*((x(m, n) – ͞x_3_ )^3^ + ((y(m, n) – ͞y_3_ )^3^)* | Mass skewness for 3^rd^ largest segment | Objects | 2 |
| *f_102_* | 0.453 | $\frac{1}{MN}\sum_{k=1}^{R}$*I* $\left( \frac{\text{area(}\text{p}\text{k}\text{)}}{\text{area(}\text{g}\text{k}\text{)}}> \delta\right)$ ** \|area(p_k_)\|* | Shape convexity feature | Objects | 2 |
| *f_103_* | 0.360 | ${}_{i,j\in\{1, \ldots, 5\}}^{\max}{}$*(\|\|h(i) – h(j)\|\|_al_)* | Hue contrast between segments | Objects (Color) | 2 |
| *f_104_* | 0.524 | ${}_{i,j\in\{1, \ldots, 5\}}^{\max}{}$*(\|s(i) – s(j)\|)* | Saturation contrast between segments | Objects (Color) | 2 |
| *f_105_* | 0.509 | ${}_{i,j\in\{1, \ldots, 5\}}^{\max}{}$*(\|l(i) – l(j)\|)* | Brightness contrast between segments | Objects (Color) | 2 |
| *f_106_* | 0.332 | ${}_{i,j\in\{1, \ldots, 5\}}^{\max}{}$*(\|b(i) – b(j)\|)* | Blur contrast between segments | Objects | 2 |
| *f_107_* | 0.167 | $\frac{\sum_{(m, n)\in M6 M7 M10 M11} (C{}_{3}^{H}{}(m, n) + C{}_{3}^{V}{}(m, n) + C{}_{3}^{D}{}(m, n))}{\sum_{i=1}^{16} \sum_{(m, n)\in Mi} (C{}_{3}^{H}{}(m, n) + C{}_{3}^{V}{}(m, n) + C{}_{3}^{D}{}(m, n))}$ | Low depth of field indicator for hue (HSV) | Texture | 1 |
| *f_108_* | 0.103 | $\frac{\sum_{(m, n)\in M6 M7 M10 M11} (C{}_{3}^{H}{}(m, n) + C{}_{3}^{V}{}(m, n) + C{}_{3}^{D}{}(m, n))}{\sum_{i=1}^{16} \sum_{(m, n)\in Mi} (C{}_{3}^{H}{}(m, n) + C{}_{3}^{V}{}(m, n) + C{}_{3}^{D}{}(m, n))}$ | Low depth of field indicator for saturation (HSV) | Texture | 1 |
| *f_109_* | 0.068 | $\frac{\sum_{(m, n)\in M6 M7 M10 M11} (C{}_{3}^{H}{}(m, n) + C{}_{3}^{V}{}(m, n) + C{}_{3}^{D}{}(m, n))}{\sum_{i=1}^{16} \sum_{(m, n)\in Mi} (C{}_{3}^{H}{}(m, n) + C{}_{3}^{V}{}(m, n) + C{}_{3}^{D}{}(m, n))}$ | Low depth of field indicator for value (HSV) | Texture | 1 |
