# Supplementary material for: Can we measure beauty? Computational evaluation of coral reef aesthetics
Source: PeerJ. 2015 Nov 10;3:e1390. doi: 10.7717/peerj.1390 (PMC4647610; doi:10.7717/peerj.1390)
Supplement: Table S2 — Overview of sampling locations with coordinates along with their respective NCEAS scores the number of photographic images used from each location and their calculated coral reef aesthetic value. Tukey connecting letters report indicates sites with significant difference in their coral reef aesthetic value. [file peerj-03-1390-s005.docx]

| **Region** | **Site** | **# images** | **NCEAS score** | **Coral reef aesthetic value** | **Tukey** | **Latitude** | **Longitude** |
| --- | --- | --- | --- | --- | --- | --- | --- |
| Caribbean | Barbuda | 306 | 49.39 | 46.49 ± 0.23 | A | 17.532 | -61.75 |
| Caribbean | Curacaobuoy2 | 243 | 49.19 | 45.15 ± 0.26 | B | 12.124 | -68.973 |
| Caribbean | CuracaoSeaquarium | 244 | 31.95 | 31.71 ± 0.25 | C | 12.081 | -68.894 |
| Caribbean | KleinCuracao | 114 | 13.07 | 18.05 ± 0.37 | F, G | 11.978 | -68.643 |
| Pacific | Kiritimati | 219 | 30.06 | 28.10 ± 0.27 | D | 1.929 | -157.545 |
| Pacific | Ant atoll | 134 | 24.39 | 23.25 ± 0.34 | E | 6.745 | 157.957 |
| Pacific | Fanning | 229 | 19.48 | 18.89 ± 0.26 | F | 3.804 | -159.339 |
| Pacific | Ant atoll | 33 | 15.16 | 20.24 ± 0.69 | F | 6.811 | 157.969 |
| Pacific | Ant atoll | 107 | 14.11 | 16.74 ± 0.38 | G | 6.801 | 157.962 |
| Pacific | Palmyra | 238 | 7.26 | 9.37 ± 0.26 | H | 5.897 | -162.047 |
| Pacific | Kingman | 250 | 3.65 | 6.65 ± 0.25 | I | 6.445 | -162.372 |
